# The Baltimore College of Dental Surgery

**Published:** 1894-04

**Authors:** 


THE BALTIMORE COLLEGE OF DENTAL SURGERY.—The fifty-fourth Annual Commencement exercises were held at the Lyceum Theatre, March 20th, 1894, at 8 P. M.

The announcement of the graduates was made by Prof. M. Whilldin Foster, M. D., D. D. S., owing to the illness of the Dean, Prof. R. B. Winder.

The address to the graduates was delivered by Rev. Robert H. Payne, of Mt. Calvary P. E. Church, and the valedictory by E. Hoffmeister, PH. D.

The degree of "Doctor of Dental Surgery" was conferred upon the following gentlemen :

Bernard Bar, Md.; Thomas Edward Black, N. C.; Ellery Percival Blanchard, Maine; Emmet; Jos. Collins, Ga.; Caleb Dorsey, Md.; Adelard Jos. Fortier, R. I.; Wilbert Ennis Foster, Texas; Archie Russell Gardner, Mont.; Frederick England Hawkesworth, Canada; Edw. Hoffmeister, Ph. D., Md.; Chas Hutchinson, Va.; Arthur Jay Kiser, Ohio; Almus Owen, Texas; David Burdett Payne, N. Y.; Elton Perry, Texas; Wm. Lewis Peters, Mass.; Winston Stephens, Fla.; Geo. Everett Thweatt, Va.; Robert Sinclair Corse, Jr., Md.; Jas. Horton Cowen, N. Y.; Frederick North Damon, Mass.; Wm. Preston Davis, Miss.; Thos. Mauck Guier, Md.; Byron Foster Hadley, Mass.; Alfred Chas. Hafke, D. D. S., Germany; Wm. Sprigg Hamilton, Ga.; Louis Klingelhoffer, Ohio; C. Herman Kopperl, Texas; Chas. Bruce McCue, Md.; Wm. Gaston Mizell, Jr., N. C.; David Saunders Ray, N. C.; Nicholas Edgar Rierson, N. C.; Jno. Geo. Schmetzer, S. C.; George Ezra Shattuck, Miss.; Chas.

Richard Twilley, Md., Wm. Frederick Walz, Va.; Daniel Bitner Williams, Pa.

The Diamond Medal of Faculty was awarded to D. B. Williams, of Penna.

The Gold Medal of James Hart was awarded to F. E. Hawkesworth, of Canada.

The Snowden & Cowman Prize was awarded to E. Perry, of Texas.

The S. S. White Prize, Dental Engine, was awarded to A. J. Kiser, of Ohia.

The Farrar Prize was awarded to F. E. Hawksworth, of Canada.

The S. S. White Varney Plugger Prize was awarded to E. P. Blanchard, of Maine.

